# TMEM158 promotes the proliferation and migration of glioma cells via STAT3 signaling in glioblastomas

**DOI:** 10.1038/s41417-021-00414-5

**Published:** 2022-01-06

**Authors:** Jiabo Li, Xuya Wang, Lulu Chen, Jinhao Zhang, Yiming Zhang, Xiao Ren, Jinzhang Sun, Xiaoguang Fan, Jikang Fan, Tao Li, Luqing Tong, Li Yi, Lei Chen, Jie Liu, Guanjie Shang, Xiude Ren, Hao Zhang, Shengping Yu, Haolang Ming, Qiang Huang, Jun Dong, Chen Zhang, Xuejun Yang

**Affiliations:** 1 Department of Neurosurgery, Tianjin Medical University General Hospital, Tianjin, China; 2 Laboratory of Neuro-oncology, Tianjin Neurological Institute, Tianjin, China; 3 Department of Neurosurgery, The First Affiliated Hospital, Zhejiang University School of Medicine, Hangzhou, Zhejiang China; 4 Department of Neurosurgery, Union Hospital, Tongji Medical College, Huazhong University of Science and Technology, Wuhan, Hubei China; 5 Department of Neurosurgery, The Second Affiliated Hospital of Soochow University, Suzhou, Jiangsu China

**Keywords:** CNS cancer, Oncogenes, CNS cancer, Tumour biomarkers

## Abstract

Glioblastoma is the most common primary intracranial malignant tumor in adults and has high morbidity and high mortality. TMEM158 has been reported to promote the progression of solid tumors. However, its potential role in glioma is still unclear. Here, we found that TMEM158 expression in human glioma cells in the tumor core was significantly higher than that in noncancerous cells at the tumor edge using bioinformatics analysis. Cancer cells in patients with primary GBMs harbored significantly higher expression of TMEM158 than those in patients with WHO grade II or III gliomas. Interestingly, regardless of tumor grading, human glioma samples that were IDH1-wild-type (IDH1-WT) exhibited higher expression of TMEM158 than those with IDH1-mutant (IDH1-Mut). We also illustrated that TMEM158 mRNA expression was correlated with poor overall survival in glioma patients. Furthermore, we demonstrated that silencing TMEM158 inhibited the proliferation of glioma cells and that TMEM158 overexpression promoted the migration and invasion of glioma cells by stimulating the EMT process. We found that the underlying mechanism involves STAT3 activation mediating TMEM158-driven glioma progression. In vivo results further confirmed the inhibitory effect of the TMEM158 downregulation on glioma growth. Collectively, these findings further our understanding of the oncogenic function of TMEM158 in gliomas, which represents a potential therapeutic target, especially for GBMs.

## Introduction

Glioblastoma (GBM) is the most common and aggressive primary central nervous system (CNS) malignant tumor in adults, accounting for ~48.6% of all malignant CNS tumors [1]. Although the current standard treatment of GBM includes safe resection of tumor to the maximum extent or biopsy, followed by administration of temozolomide (TMZ) combined with radiotherapy and maintenance TMZ for 6–12 months [2], the median survival time of GBM patients is only 14–16 months, and the 5-year overall survival (OS) remains <10% [3]. Although management of GBM frequently involves the use of TMZ, due to severe hematological toxicity and low blood-brain barrier permeability, GBM treatment requires more breakthroughs.

During the last decade, new clinical trials for GBM have continuously emerged, including targeted drug therapy (such as EGFR [4], BRAF^V600E^ [5], MET [6], and immune checkpoint inhibitors (PD-1/PD-L1 [7–9], CTLA4 [10])), vaccination [11, 12], virotherapy [13–15], and CAR T-cell [16]. However, most of these trials have failed to prolong the OS of GBM patients in phase I–III clinical trials. Encouragingly, one phase III clinical trial of Tumor-Treating Fields (TTFields) for newly diagnosed GBM recruited 695 patients, and the results showed that patients treated with TMZ plus TTFields exhibited significantly longer overall survival (OS, 20.9 months vs. 16.0 months) and progression-free survival (6.7 months vs. 4.0 months) than those treated with TMZ alone [3]. Therefore, additional targeted molecules that are important for glioma progression need to be discovered.

Transmembrane protein 158 (TMEM158), also known as RIS1, P40BBP, BBP, and HBBP, a member of the TMEM family, was first confirmed as an upregulated candidate tumor suppressor gene during Ras-induced senescence in haploid fibroblasts infected with RAS V12 lentivirus [17]. The expression level and biological mechanisms of TMEM158 have since been reported in many tumors [18–21]. Mohammed Ael et al. identified TMEM158 as a powerful predictive marker for cisplatin therapy efficacy in non-small cell lung cancer [18]. In addition, TMEM158 is upregulated in ovarian cancer and significantly promotes the proliferation, invasion, cell adhesion, and tumorigenesis of ovarian cancer cells [19]. TMEM158 was also found to be significantly overexpressed in pancreatic cancer and is associated with larger tumor size and poorer prognosis. Knockdown of TMEM158 inhibits the proliferation, migration, and invasion of pancreatic cancer cells [20]. Moreover, loss of function of TMEM158 significantly decreased the proliferation and migration of colorectal cancer cells and inhibited multidrug resistance [21]. However, the roles of TMEM158 in glioma have not been explored.

Signal transducer and activator of transcription 3 (STAT3) may be the key point of multiple major oncogenic signaling pathways, such as epithelial growth factor receptor (EGFR), c-MET, and Janus family of kinases (JAK) [22, 23]. STAT3 is persistently activated in nearly 70% of human cancers [24], especially in gliomas [25]. Previous studies have shown that excessive activation of the STAT3 signaling pathway is involved in proliferation, metastasis, angiogenesis, stemness, therapeutic resistance, and the immunosuppressive microenvironment in GBM [26–28]. As a key molecule, activation of STAT3 promote proliferation, migration, and epithelial-mesenchymal transition (EMT) of glioma cells [29]. However, there is currently no comprehensive analysis of the relationship between TMEM158 and the STAT3 signaling pathway.

In this study, we first investigated the clinicopathological and biological characteristics of TMEM158 in gliomas. The clinicopathological features that were evaluated included WHO grade, IDH mutation and 1p/19q status, gliomas tissue microarray, and OS. In addition, the effects of TMEM158 loss- and gain-of-function on the proliferation, migration, and invasion of glioma cells were assessed. Mechanistically, we further confirmed that TMEM158 enhanced glioma cells proliferation, migration, and invasion as well as the progression of EMT by activating STAT3 signaling. Importantly, TMEM158 downregulation inhibited glioma cell growth and decreased the expression of p-STAT3 and Ki-67 in vivo. Taken together, our findings revealed that TMEM158 is upregulated in GBM and promotes GBM tumorigenesis and aggressiveness by activating the STAT3 signaling pathway, highlighting that it may represent an effective therapeutic target in GBM.

## Materials and methods

### Bioinformatics analysis

The Cancer Genome Atlas (TCGA) pan-cancer RNA-seq data (level 3) and GTEx RNA-seq data were obtained from the UCSC website (https://xenabrowser.net/datapages/), and the datasets were used by the UCSC team to remove batch effects and perform transcripts per million conversion. TMEM158 gene expression and clinical data were obtained from TCGA database (https://cancergenome.nih.gov) and the Chinese Glioma Genome Atlas (CGGA) database (http://www.cgga.org.cn). Kaplan–Meier survival curves were used to analyze differences in TMEM158 expression and their effects on OS in glioma patients. Differentially expressed genes (DEGs) were screened with R language using the “DESeq2” package. Then, GSEA of the HALLMARK gene set was performed based on the Log2FoldChange value ranking of the DEGs by R language using the “clusterProfiler” package. We also performed Gene Ontology (GO) analyses and Kyoto Encyclopedia of Genes and Genomes (KEGG) pathway enrichment analyses using the online DAVID database (https://david.ncifcrf.gov).

### Clinical sample collection

Clinical glioma tissues and glioma pathologic diagnoses were obtained from the Department of Neurosurgery, Tianjin Medical University General Hospital, China, from August 2011 to April 2017 [26]. All samples were histologically diagnosed by pathologists based on the World Health Organization (WHO) classification for brain tumors. Written informed consent was obtained from all donors or their relatives. This study was performed in accordance with the principles of the Helsinki Declaration and was approved by the ethical committee of Tianjin Medical University General Hospital.

### Tissue microarray (TMA)

The TMA was run using tissues from 55 patients, including 2 nontumor cases (temporal lobe epilepsy and cortical dysplasia), 1 WHO I case, 12 WHO II cases, 12 WHO III cases, and 28 WHO IV cases. For each tumor mass, we divided the tissues into intratumor, tumor border, and peritumor tissues. In this study, we excluded WHO I cases and only analyzed intratumoral and peritumoral staining.

### H&E staining and immunohistochemistry (IHC) analysis

Paraffin-embedded tissues used for H&E staining and IHC analysis were prepared as previously described [30, 31]. Glioma tissues and mouse brain tissues were incubated with primary antibodies (TMEM158, 1:100, ab98335, Abcam, USA; p-STAT3, 1:100, 9145S, Cell Signaling Technology, USA; and Ki-67, 1:100, 9027S, Cell Signaling Technology, USA), IHC markers were detected using a goat anti-rabbit IgG two-step detection kit (PV-9000, ZSGB-Bio, China). Next, the slides were counterstained with Mayer Hematoxylin Solution (G1080, Solarbio, China) for nuclear staining. The IHC staining images were acquired a VANOX microscope (Olympus, Japan). The intensity score was graded as 0 (negative), 1 (weakly positive, light brown), 2 (moderately positive, brown), or 3 (strongly positive, dark brown). The quantity score was graded as 0 (negative), 1 (≤25%), 2 (26–50%), 3 (51–75%), or 4 (>75%). The total IHC staining score was calculated by adding the intensity and quantity scores.

### Cell lines and cell culture

Human glioma A172, LN18, LN229, and T98G cell lines were purchased from ATCC (USA). SNB19, U251MG, and U87MG cells were purchased from the Chinese Academy of Sciences Cell Bank (China). The human glioma TJ905 cell line was isolated from human GBM tissue and cultured in DMEM/F12 medium (Gibco, USA) supplemented with 10% fetal bovine serum (FBS, Gibco, USA). All other glioma cell lines were cultured in DMEM (Gibco, USA) supplemented with 10% FBS and incubated in 5% CO_2_ at 37 °C.

### Lentivirus and plasmid transfection

We constructed shRNA-TMEM158 sequences (sh-1, 5′-ccTGCCCAACGGCATGGAACA-3′; sh-2, 5′-gcATTTCTGCTGCCTAGACTT-3′) using the GV493 vector, and a scramble sequence (5′-TTCTCCGAACGTGTCACGT-3′) was designed as a negative control. We also constructed a TMEM158-overexpressing plasmid using the GV492 vector (GeneChem, China). Lentiviral transfection was conducted according to the manufacturer’s manual. After infection, U87MG, U251MG, and TJ905 cells were selected using 2.00 μg/ml puromycin solution. Plasmids were purchased from Hanbio (China). A STAT3-overexpressing plasmid was constructed using the pcDNA3.1 vector. Plasmids were transiently transfected into cells using Lipofectamine 3000 (Invitrogen, US).

### RNA isolation and real-time polymerase chain reaction (RT-PCR)

Total RNA from cells or tissues was extracted using TRIzol reagent (15596018, Thermo Fisher Scientific, USA), and RNA (5 µg) was reverse transcribed to cDNA using the GoScript Reverse Transcription System (A5001, Promega, USA). RT-PCR was conducted as previously described [32]. Expression of TMEM158 and GAPDH (internal control) mRNA was detected using GoTaq qPCR Master Mix (A6001, Promega, USA). The primer sequences (Genewiz, China) were as follow: TMEM158 Forward: 5′-CGCTTCCAGTTCCGAAAAGC-3′, Reverse: 5′-GCAGGGGGATGCAATAGAGG-3′; GAPDH Forward: 5′-GGTGGTCTCCTCTGACTTCAACA-3′, Reverse: 5′-GTTGCTGTAGCCAAATTCGTTGT-3′. Data were analyzed using the relative standard curve method and normalized to GAPDH.

### Cell counting kit-8 assay (CCK-8)

Glioma cell viability in response to TMEM158 loss- and gain-of-function and rescue experiments was measured using CCK-8 (CK04, DOJINDO, China) according to the manufacturer’s protocol. Glioma cells were seeded into 96-well plates at 2.0 × 10^3^ cells per well and incubated in 5% CO2 at 37 °C for 1, 2, 3, and 4 day. Then, the cells were incubated with CCK-8 solution for 1 h, and the absorbance was measured at 450 nm using a microplate luminometer (BioTek, USA).

### Colony-formation assay

Glioma cells were seeded into six-well plates and incubated in 5% CO_2_ at 37 °C for 14 day. Then, the cells were washed with PBS, fixed in 4% paraformaldehyde (P1110, Solarbio, China), and stained with 2.5% crystal violet stain solution (G1061, Solarbio, China). The colony-forming efficiency was defined as the ratio of the number of colonies formed to the number of cells seeded.

### Transwell assay

Cells (1.0 × 10^4^ cells) were seeded in serum-free DMEM or DMEM/F12 into the upper part of the Cell Culture Insert (24-well format; 353097, Corning, USA) for Transwell migration assay, and the lower chamber was filled with medium supplemented with 10% FBS as a chemoattractant. For the Transwell invasion assay, first, Matrigel was added to the bottom of the chamber. Cells (5.0 × 10^4^ cells) were then seeded in serum-free DMEM or DMEM/F12 into the upper chamber. After incubation for 24 h, the noninvading cells in the upper chamber were removed using cotton swab. Transwell chambers containing the invading cells were fixed with 4% paraformaldehyde and stained with 2.5% crystal violet stain solution. The number of invading cells on the lower surface of the filters was then quantified.

### Western blotting

Cells were lysed in RIPA buffer (R0010, Solarbio, China) containing PMSF (dilution, 1:100; P0100, Solarbio, China), protein phosphatase inhibitor (dilution, 1:100; P1260, Solarbio, China), and protease inhibitor mixture (dilution, 1:100; P6730, Solarbio, China). Then, the total protein concentration was measured using a BCA Protein Assay Kit (PC0020, Solarbio, China) according to the manufacturer’s instructions. Subsequently, 30 µg of each protein sample was analyzed. Sodium dodecyl sulfate-polyacrylamide gel electrophoresis was used to separate the extracted proteins. Then, the proteins were blotted onto PVDF transfer membranes (ISEQ00010, Millipore, USA), and blocked with 5% skimmed milk. Next, the PVDF membranes were incubated overnight at 4 °C with the primary antibodies. Then, the membranes were incubated with goat anti-rabbit/mouse IgG secondary antibody (dilution, 1:3000; ZB-2301, ZB-2305, ZSGB-BIO, China) for 1 h at room temperature. Protein expression was analyzed using GBOX (Syngene Company, UK) and a chemiluminescent HRP substrate (WBKLS0500, Millipore, USA). The primary antibodies used in this study targeted the following proteins (dilution, 1:1000): TMEM158 (ab98335) was obtained from Abcam (UK); E-cadherin (E-Ca; 3195S), N-cadherin (N-Ca; 13116S), vimentin (5741S), Snail (3879S) and p-STAT3 (9145S) were purchased from Cell Signaling Technology (USA); STAT3 (A11867) was obtained from ABclonal (China); and β-actin (TA-09) was purchased from ZSGB-BIO (China).

### Intracranial xenograft model in nude mice

All animal experiments were approved by the Ethical Committee of the Tianjin Medical University General Hospital. An intracranial xenograft mouse model was established as previously described [31]. U251MG cells were stably transfected with sh-TMEM158 and OE-TMEM158 lentivirus, and control cells were injected into the brains of mice. Tumor burden was monitored by bioluminescence imaging every week starting on day 7 after implantation using an IVIS Spectrum Live Imaging System (Perkin Elmer, USA). Body weight and OS of mice in both groups were monitored every day. The brains of the mice were carefully extracted when the mice died, fixed in 10% formalin, and embedded in paraffin for H&E staining and IHC staining.

### Statistics

All experimental data were examined at least three times. Statistical analysis was performed using SPSS 20. All quantitative data are presented as the mean ± SD. Survival analyses were conducted using the log-rank (Mantel–Cox) test in GraphPad Prism 8.01. An unpaired *t*-test was used to compare the means of two groups, and a two-tailed *p* value of <0.05 was considered statistically significant.

## Results

### TMEM158 mRNA is highly expressed in IDH1-WT GBM

**Fig. 1:**
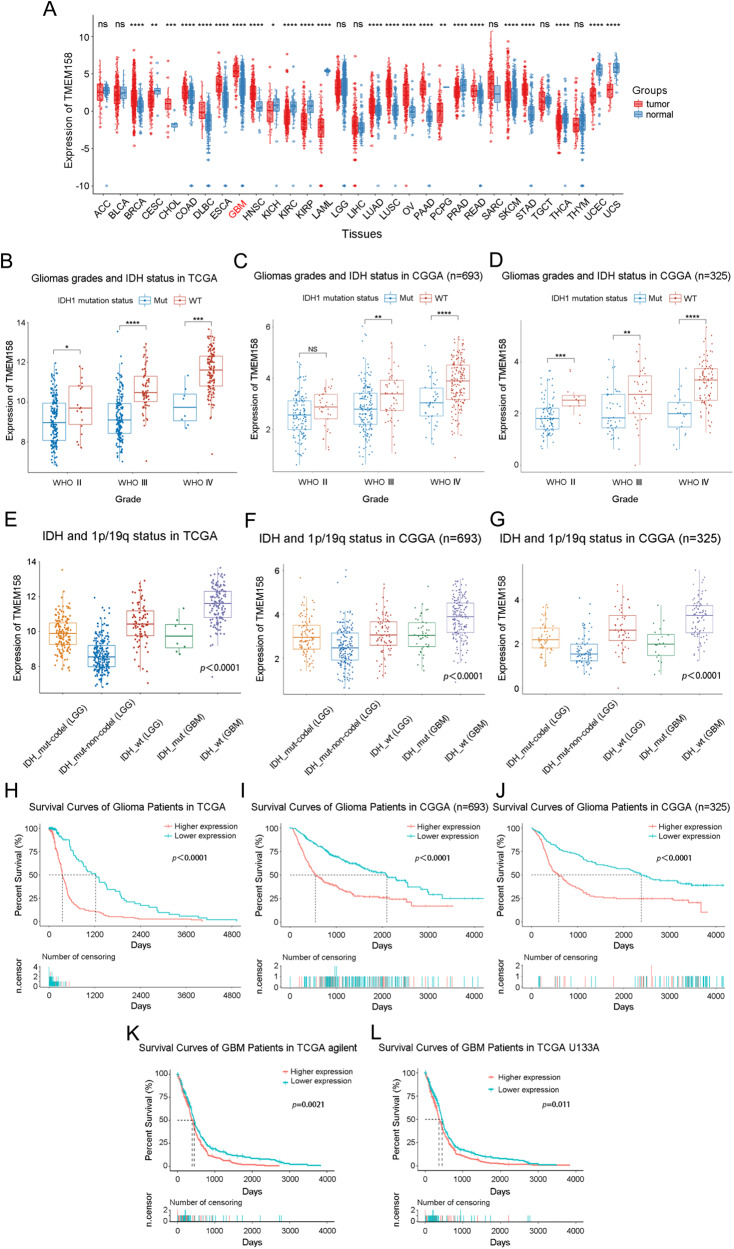
TMEM158 mRNA is highly expressed in IDH1-WT GBMs and is correlated with poor prognosis in glioma patients. **A** Expression profile of TMEM158 in 31 kinds of cancers and their paired normal tissues from TCGA database. **B**–**D** Relationship between TMEM158 mRNA expression and WHO glioma grades and IDH1 mutation status of glioma samples in the TCGA and CGGA databases. **E**–**G** Expression of TMEM158 in IDH1-Mut-codel LGG, IDH1-Mut-noncodel LGG, IDH1-WT LGG, IDH1-Mut GBM, and IDH1-WT GBM. **H**–**J** TCGA and CGGA datasets were used for survival analysis of the two groups of glioma patients with higher TMEM158 expression and lower TMEM158 expression in glioma patients. **K**–**L** Kaplan–Meier survival curves were used to analyze the overall survival of GBM patients with higher expression of TMEM158 and lower expression of TMEM158 in the TCGA Agilent and U133A databases. (^ns^*p* > 0.05, *p < 0.05, **p < 0.01, ***p < 0.001, ****p < 0.0001).

First, we analyzed the expression profile of TMEM158 across cancers. TMEM158 expression values in 33 types of cancers were extracted from TCGA database and compared to TMEM158 expression values in tissues from non-lesion sites obtained from GTEx database (Fig. 1A). Among 33 cancers, mesothelioma (MESO) and uveal melanoma did not match their corresponding normal tissue. In the remaining 31 cancers, there were no statistically significant difference in TMEM158 expression in 7 cancers (adrenocortical carcinoma, ACC; bladder urothelial carcinoma, BLCA; lower grade glioma, LGG; liver hepatocellular carcinoma, LIHC; sarcoma, SARC; testicular germ cell tumors, TGCT; thymoma, THYM). TMEM158 expression was significantly downregulated in 10 cancers (cervical squamous cell carcinoma and endocervical adenocarcinoma, CESC; kidney chromophobe, KICH; kidney renal clear cell carcinoma, KIRC; kidney renal papillary cell carcinoma, KIRP; acute myeloid leukemia, LAML; pheochromocytoma and paraganglioma, PCPG; prostate adenocarcinoma, PRAD; thyroid carcinoma, THCA; uterine corpus endometrial carcinoma, UCEC; uterine carcinosarcoma, UCS), and significantly upregulated in 14 cancers (breast invasive carcinoma, BRCA; cholangiocarcinoma, CHOL; colon adenocarcinoma, COAD; lymphoid neoplasm diffuse large B-cell lymphoma, DLBC; esophageal carcinoma, ESCA; glioblastoma multiforme, GBM; head and neck squamous cell carcinoma, HNSC; lung adenocarcinoma, LUAD; lung squamous cell carcinoma, LUSC; ovarian serous cystadenocarcinoma, OV; pancreatic adenocarcinoma, PAAD; rectum adenocarcinoma, READ; skin cutaneous melanoma, SKCM; stomach adenocarcinoma, STAD), especially in GBM (GBM: GTEx = 5.38: 3.54, *p* < 0.0001. LGG: GTEx = 3.32: 3.54, *p* > 0.05) (Fig. 1A).

Furthermore, we performed a stratified analysis based on the WHO classification and IDH1 mutation status of glioma samples in the TCGA and CGGA databases. We found that TMEM158 mRNA expression increased with tumor grade, and its expression in IDH1-wild-type (IDH1-WT) gliomas of each grade was higher than that in IDH1-mutant (IDH1-Mut) gliomas. Importantly, TMEM158 exhibited the highest expression level in IDH1-WT GBM (Fig. 1B–D). Adding 1p/19q status analysis, we found that the status of 1p/19q was also correlated with TMEM158 mRNA expression levels. Compared to lower grade gliomas with IDH1 mutation and non-1p/19q codeletion (IDH1-Mut-noncodel, LGG), TMEM158 mRNA expression was higher in lower grade gliomas with IDH1 mutation and 1p/19q codeletion (IDH1-Mut-codel, LGG) (Fig. 1E–G). These results suggested that TMEM158 may be preferentially expressed in oligodendrogliomas. Moreover, we found that TMEM158 mRNA expression levels in IDH1-WT LGGs were only inferior to that in IDH1-WT GBMs and were higher than that in any other type, including IDH-Mut GBMs (Fig. 1E–G). These results suggest that TMEM158 is closed related to IDH1 status and is preferentially expressed in IDH1-WT GBMs.

### Upregulated TMEM158 expression is correlated with poor prognosis in glioma patients

To determine the prognostic value of TMEM158 gene expression in glioma patients, Kaplan–Meier (K–M) survival curves were performed using data from the TCGA and CGGA clinical information, RNA-seq, and microarray datasets. The results showed that the OS time of glioma patients with higher TMEM158 expression was shorter than that of glioma patients with lower TMEM158 expression in the TCGA RNA-seq database (*p* < 0.0001) (Fig. 1H). Moreover, in the CGGA RNA-seq database (*n* = 693 and *n* = 325), glioma patients with higher TMEM158 expression had a worse prognosis than those with lower TMEM158 expression (*p* < 0.0001) (Fig. 1I–J). Importantly, GBM patients with higher expression of TMEM158 also had a worse prognosis than those with lower expression of TMEM158 in the TCGA agilent dataset (*n* = 488) and TCGA U133A dataset (*n* = 525) (TCGA agilent dataset: *p* = 0.0021; TCGA U133A dataset: *p* = 0.011) (Fig. 1K–L). These results indicate that TMEM158 may be a prognostic factor for glioma patients.

### TMEM158 is related to glioma grades and was preferentially expressed in the core of glioma tissues

**Fig. 2:**
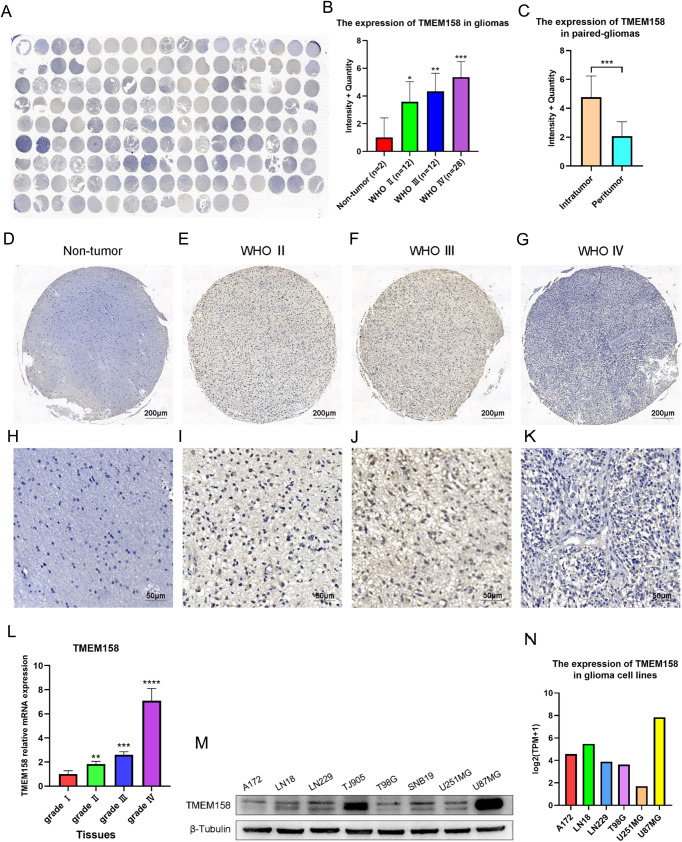
TMEM158 is related to glioma grade and is preferentially expressed in the core of glioma tissues. **A** TMEM158 protein expression was detected in a tissue microarray (TMA) (nontumor, *n* = 2; WHO grade II, *n* = 12; WHO grade II, *n* = 12; WHO grade IV, *n* = 28). **B** Total IHC staining score of TMA was related to WHO glioma grades, and nontumor samples exhibited the lowest protein expression of TMEM158. **C** The intratumoral and peritumoral expression patterns of TMEM158. **D**–**G** Representative image of TMEM158 expression on nontumor, WHO II, WHO III, and WHO IV tumor samples. **H**–**K** Partial enlarged image of (**D**–**G**). **L** RT-PCR results show the expression of TMEM158 mRNA in different grades of gliomas. **M** Western blotting was used to determine expression of TMEM158 in 8 different glioma cell lines. **N** Expression of TMEM158 in glioma cell lines in the CCLE database. (^ns^*p* > 0.05, *p < 0.05, **p < 0.01, ***p < 0.001, ****p < 0.0001).

To further explore the expression pattern of TMEM158 in gliomas, we used IHC and RT-PCR to determine the relationship between the expression of TMEM158 in glioma tissue and glioma grade. Therefore, we took advantage of clinical human glioma samples to detect the protein pattern of TMEM158 in a tissue microarray (TMA) (Fig. 2A). The TMA data (nontumor, *n* = 2; WHO grade II, *n* = 12; WHO grade III, *n* = 12; WHO grade IV, *n* = 28) revealed that TMEM158 expression was correlated with malignancy classified using the WHO system (Fig. 2B, D–K). Expression of TMEM158 was abundant in WHO grade III (Fig. 2F, J) and IV glioma tissues (Fig. 2G, K). However, it was hardly detectable in nontumor brain tissues (Fig. 2D, H) or WHO grade II glioma tissues (Fig. 2E, I). The RT-PCR results for glioma tissues also verified that TMEM158 mRNA was highest in WHO grade IV glioma tissues and lowest in WHO grade I glioma tissues (Fig. 2L). Moreover, a higher WHO grade was associated with higher TMEM158 expression (Table 1). Interestingly, in 27 paired glioma tissues, the IHC staining total score was higher in intratumoral tissues than in peritumoral tissues (Fig. 2C). Overall, these data suggest that TMEM158 is associated with glioma grades and is preferentially expressed in intratumoral of glioma tissues.

**Table 1:** The clinicopathological relevance analysis of TMEM158 expression in patients with gliomas.

Variable	Total	TMEM158 expression		*p* value
		High	Low	
Age				0.157
≤45 years	19	12	7	
>45 years	33	27	6	
Gender				0.510
Male	26	19	7	
Female	26	21	5	
WHO grade				0.003**
LGG	24	14	10	
GBM	28	26	2	

**p < 0.01.

Furthermore, we assessed the expression of TMEM158 in eight different glioma cell lines, including A172, LN18, LN229, TJ905, T98G, SNB19, U251MG, and U87MG, using western blotting, among which TJ905 is a primary GBM cell line. The result showed that TMEM158 exhibited the highest expression in U87MG cells and had a lower expression in U251MG cells (Fig. 2M). The results of the CCLE database also verified that TMEM158 is highly expressed in U87MG cells and expressed at lower levels in U251MG cells (Fig. 2N). Therefore, we selected the U87MG, U251MG, and TJ905 cell lines to further explore the function of TMEM158 in GBM.

### TMEM158 overexpression promoted the proliferation, migration, and invasion of glioma cells

**Fig. 3:**
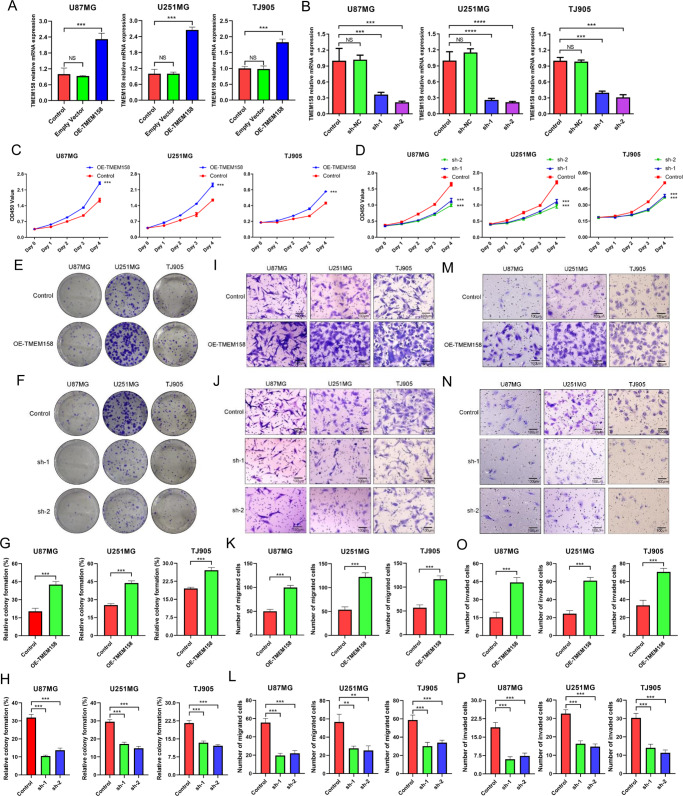
TMEM158 overexpression promotes the proliferation, migration, and invasion of glioma cells. **A**–**B** RT-PCR results showing the transfection efficiency of lentiviral overexpression (OE-TMEM158) and knockdown (shRNA-TMEM158-1, sh-1; shRNA-TMEM158-2, sh-2) and the expression of TMEM158 in U87MG, U251MG, and TJ905 glioma cell lines. **C**–**D** CCK-8 results showing the proliferation of U87MG, U251MG, and TJ905 glioma cells in response to upregulating and downregulating TMEM158 expression. **E**, **G** Colony-formation assay results showing the colony-formation efficiency of glioma cells after overexpression of TMEM158. **F**, **H** Colony-formation assay results showing the colony formation efficiency of glioma cells after silencing TMEM158 expression. **I**–**L** The migration ability in U87MG, U251MG, and TJ905 glioma cells in response to overexpression and knockdown of TMEM158 was detected by Transwell migration assay. **K** and **L** Show the statistical histogram of the quantification of migrated cells after upregulation and downregulation of TMEM158, respectively. **M**–**P** The representative images of Transwell invasion assay for glioma cell lines after gain- and loss-of-function TMEM158. The number of invaded cells is shown in (**O** and **P**). Data in (**K**, **L**, **O**, and **P**) are shown as mean ± SD. Scale bar = 100 μm. (^ns^*p* > 0.05, *p < 0.05, **p < 0.01, ***p < 0.001, ****p < 0.0001).

**Fig. 4:**
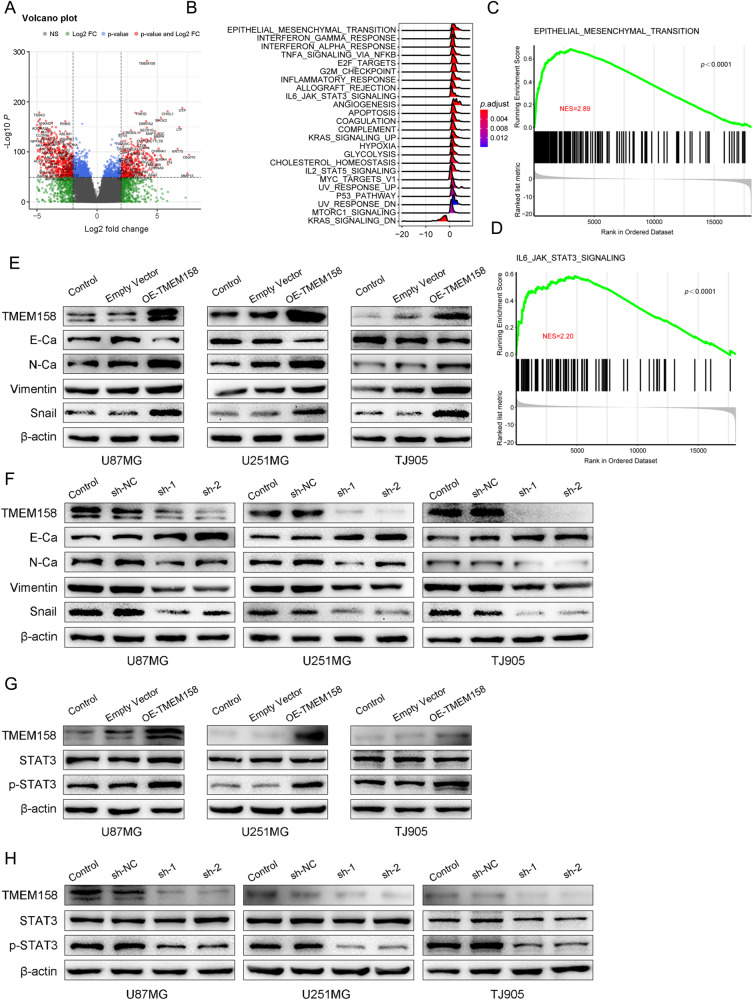
TMEM158 was positively correlated with EMT process and STAT3 signaling. **A** Volcano plot showing the differentially expressed genes (DEGs) related to TMEM158. We identified a total of 776 upregulated genes and 1040 downregulated genes. **B** GSEA and HALLMARK gene set enrichment analysis were used to enrich the pathways related to TMEM158-associated genes. **C** GSEA showed that TMEM158-associated genes were enriched in the EMT process. (NES = 2.89, *p* < 0.0001). **D** TMEM158-associated genes were enriched in the STAT3 signaling pathway by GSEA. (NES = 2.20, *p* < 0.0001) **E**–**F** Western blotting results show changes in TMEM158 and EMT marker expression (E-Ca, N-Ca, vimentin, and Snail) in U87MG, U251MG, and TJ905 glioma cells after overexpression and knockdown of TMEM158. **G**–**H** TMEM158, STAT3, and p-STAT3 protein levels in U87MG, U251MG, and TJ905 glioma cells after overexpression and knockdown TMEM158 detected by western blotting.

To explore the functions of TMEM158 in glioma cells, an overexpressing plasmid and two pairs of shRNA lentiviruses were constructed to upregulate and downregulate TMEM158 expression, respectively. We first performed RT-PCR and western blotting to determine the transfection efficiency of lentiviral overexpression (OE-TMEM158) and knockdown (shRNA-TMEM158-1, sh-1; shRNA-TMEM158-2, sh-2) and the expression of TMEM158 in U87MG, U251MG, and TJ905 glioma cell lines (Figs. 3A–B, 4E–F). Then, CCK-8 and colony-formation assay were conducted to determine the proliferation of U87MG, U251MG, and TJ905 glioma cells. As shown in Fig. 3C–D, the CCK-8 experiments demonstrated that cells transfected with OE-TMEM158 lentivirus displayed increased cell proliferation compared to the control group (*p* < 0.001) (Fig. 3C). However, cells transfected with shRNA-TMEM158 lentiviruses exhibited reduced proliferation (*p* < 0.001) (Fig. 3D). Similarly, glioma cells displayed higher colony formation efficiency after upregulating TMEM158 (Fig. 3E, G). Downregulation of TMEM158 inhibited the colony formation efficiency of glioma cells (*p* < 0.001) (Fig. 3F, H). These results indicate that TMEM158 promotes the proliferation of glioma cells.

To confirm whether TMEM158 affects the migration and invasion of glioma cells, a Transwell assay was performed. First, we used a Transwell migration assay to determine the effect of TMEM158 on the migration ability of glioma cells. The results showed that glioma cell migration ability was enhanced when TMEM158 expression was upregulated (*p* < 0.001) (Fig. 3I, K), while downregulating TMEM158 expression significantly decreased the motility of glioma cell (*p* < 0.001) (Fig. 3J, L). Next, we added Matrigel into the bottom of the chamber to conduct the Transwell invasion assay. The results also revealed that overexpression of TMEM158 expression increased the invasion ability of glioma cells (*p* < 0.001) (Fig. 3M, O). Downregulating TMEM158 expression using shRNA significantly inhibited the invasion ability of glioma cells compared to the control group (*p* < 0.001) (Fig. 3N, P). These results demonstrate that overexpression of TMEM158 significantly promotes the motility of glioma cells.

### TMEM158-associated genes in glioma are enriched in epithelial-mesenchymal transition and STAT3 signaling

To further investigate the potential mechanism of TMEM158 in gliomas, we conducted a series of bioinformatics analysis. First, we ranked the glioma samples according to their expression levels of TMEM158 in the TCGA database and then took the 25% of the samples at both ends as the high and low expression groups. Next, we used the “DESeq2” R package for selecting DEGs and used the “enhanceVolcano” R package for visualization. We obtained a total of 776 upregulated genes and 1040 downregulated genes (Fig. 4A). Furthermore, we used the “clusterProfiler” R package to perform GSEA and HALLMARK gene set enrichment analysis based on the Log2FoldChange values. The results showed that TMEM158-assiciated genes were enriched in EMT, TNFα signaling via NF-κB, IL6-JAK-STAT3 signaling, and another pathway (Fig. 4B). In addition, we performed GO analysis and KEGG pathway enrichment analysis on the TMEM158-Low-Expression genes and TMEM158-High-Expression genes, respectively. The results indicated that TMEM158 was primarily associated with the modulation of chemical synaptic transmission, extracellular matrix organization, skeletal system morphogenesis, neuroactive ligand-receptor interaction, calcium signaling pathway, and cytokine-cytokine receptor interaction (Figure S1A–D). Then, we first analyzed the TMEM158-associated genes by GSEA enrichment analysis, and the results indicated that TMEM158 was associated with the process of EMT (NES = 2.89, *p* < 0.0001) and the IL6-JAK-STAT3 signaling pathway (NES = 2.20, *p* < 0.0001) (Fig. 4C–D). Collectively, these results show that TMEM158-associated genes in gliomas are enriched in EMT and the STAT3 signaling pathway.

### TMEM158 is positively correlated with the EMT process and STAT3 signaling

Multiple studies indicate that EMT is frequently observed in invasive human cancers and plays a key role in cancer metastasis by increasing the motility of tumor cells [33, 34]. The mesenchymal subtype of GBM is related to a more aggressive and treatment-resistant phenotype and displays a poorer prognosis than other subtypes [35]. First, the GSEA results indicated that TMEM158 was related to the process of EMT (NES = 2.89, *p* < 0.0001) (Fig. 4C). Next, western blotting was conducted to confirm the function of TMEM158 in EMT. The results showed that EMT markers were significantly altered by upregulating and downregulating TMEM158 expression. E-Ca was downregulated in TMEM158-overexpressed U87MG, U251MG, and TJ905 glioma cells, while N-Ca, vimentin, and Snail were upregulated (Fig. 4E). In contrast, downregulating TMEM158 in glioma cells significantly increased the expression of E-Ca and decreased the expression of N-Ca, vimentin, and Snail (Fig. 4F). These results suggest that TMEM158 may improves the motility of glioma cells by stimulating the EMT process.

To understand the molecular mechanisms underlying TMEM158-induced proliferation, migration, and invasion of glioma cells, we examined TMEM158-associated genes, which were enriched in the IL6-JAK-STAT3 signaling pathway as assessed using HALLMARK gene set enrichment analysis (Fig. 4B). We first investigated the relationship between TMEM158 expression and the IL6-JAK-STAT3 signaling pathway via GSEA (NES = 2.20, *p* < 0.0001) (Fig. 4D). Furthermore, we performed western blotting to detect the expression of key proteins of the STAT3 signaling pathway (STAT3; phosphorylated-STAT3, p-STAT3). The results revealed that p-STAT3 was significantly increased in TMEM158- overexpressing glioma cells, whereas total STAT3 levels did not vary significantly from that of β-actin (Fig. 4G). However, reduced p-STAT3 levels were further observed in U87MG, U251MG, and TJ905 glioma cells in response to TMEM158 downregulation (Fig. 4H). These findings in this aspect indicate that TMEM158 is positively correlated with STAT3 signaling in glioma cells.

### TMEM158 mediates the proliferation, migration, and invasion of glioma cells by activating STAT3 signaling

**Fig. 5:**
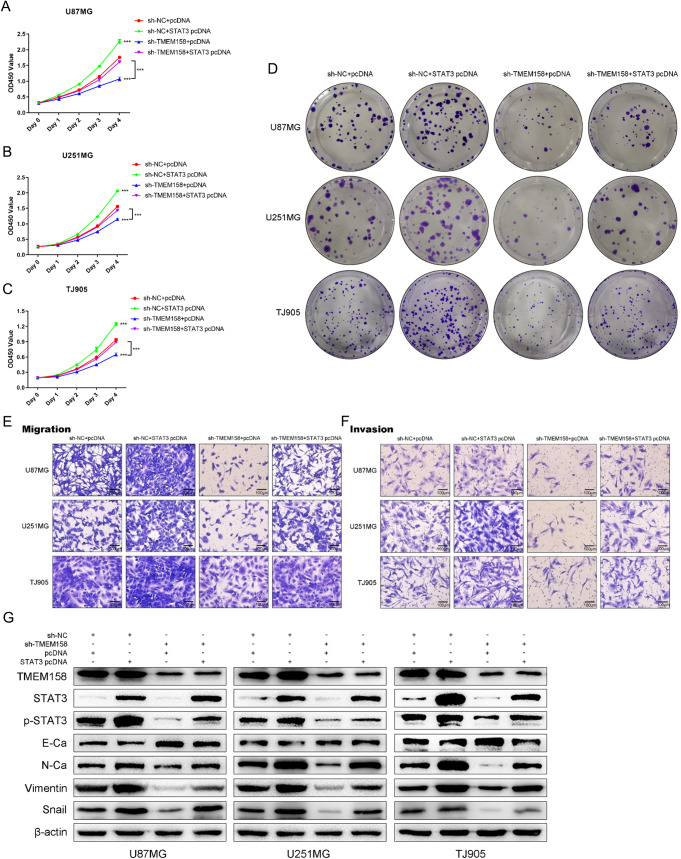
TMEM158 mediates the proliferation, migration, and invasion of glioma cells by activating STAT3 signaling. **A–C** CCK-8 results showing the proliferation of sh-NC + pcDNA, sh-NC + STAT3 pcDNA, sh-TMEM158 + pcDNA, and sh-TMEM158 + STAT3 pcDNA in the four groups of glioma cells. **D** Colony-formation assay results showing the colony formation efficiency of glioma cells after silencing TMEM158 and upregulating STAT3. **E–F** Transwell assay results showing that overexpression of STAT3 rescued the reduced migration and invasion caused by TMEM158 knockdown. **G** Western blotting results showing the expression of TMEM158, STAT3, p-STAT3, E-Ca, N-Ca, vimentin, and Snail in the four groups of glioma cells. (^ns^*p* > 0.05, *p < 0.05, **p < 0.01, ***p < 0.001, ****p < 0.0001).

To further elucidate the underlying mechanism of the involvement of STAT3 signaling in TMEM158-mediated GBM progression, we conducted a series of experiments to demonstrate whether overexpression of STAT3 by plasmid transfection rescues TMEM158 knockdown-mediated proliferation, migration, and invasion. The CCK-8 assay showed that downregulating TMEM158 expression inhibited the proliferation of glioma cells, while overexpression of STAT3 rescued the reduced proliferation caused by TMEM158 knockdown (Fig. 5A–C). The colony-formation assay also confirmed this result (Fig. 5D). Furthermore, we conducted a Transwell assay to demonstrate that upregulating STAT3 expression rescues the change in TMEM158 downregulation-mediated the migration and invasion (Fig. 5E–F). Western blotting also showed that overexpression of STAT3 reduced the expression of E-Ca and increase the expression of N-Ca, vimentin, and Snail in glioma cells in response to TMEM158 knockdown (Fig. 5G). These results indicated that TMEM158 mediates the proliferation, migration, invasion, and EMT process of glioma cells by activating STAT3 signaling.

### Silencing TMEM158 impairs the invasion of GBM cells in vivo and suppresses tumor growth

**Fig. 6:**
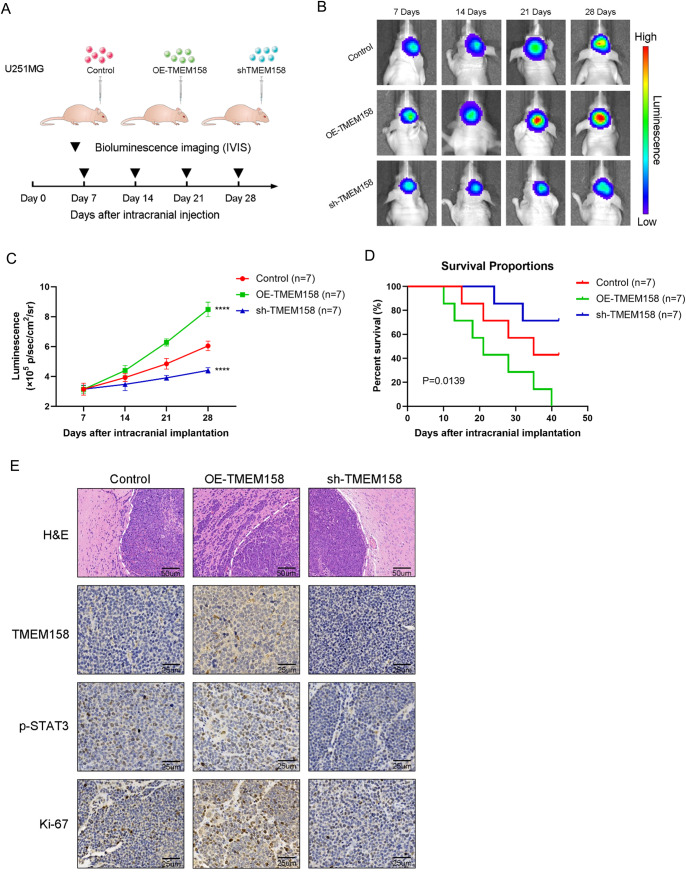
Silencing TMEM158 impairs the invasion of GBM cells in vivo and suppresses tumor growth. **A** Schematic diagram of experimental grouping, implantation, and bioluminescence imaging of the orthotopic xenograft model. **B–C** Bioluminescence imaging of tumor growth in animals. Signal intensities were quantified on days 7, 14, 21, and 28 after implantation. *n* = 7 per group. (****p < 0.0001) **D** Kaplan–Meier survival curves indicating the percentage survival of mice. **E** Representative images of H & E staining of the mouse cerebrum showing the tumor border. Immunohistochemical (IHC) staining for TMEM158, p-STAT3, and Ki67 in the samples.

To further explore the role of TMEM158 in GBM, we established the intracranial xenograft mouse model (Fig. 6A). Tumors for all three cell types were detectable by day 7. Luminescence detected in U251MG-OE-TMEM158 tumors was greater than that in control tumors and continued to increase over the course of 28 days. In contrast, U251MG-shTMEM158 tumors grew more slowly over the course of 28 days compared to control tumors (Fig. 6B–C). Kaplan–Meier survival curves demonstrated that U251MG-shTMEM158 tumor-bearing mice exhibited longer OS than the control mice. However, U251MG-OE-TMEM158 tumor-bearing mice displayed poorer survival than control U251MG tumor-bearing mice (Fig. 6D). H&E staining revealed the smaller size of the U251MG-shTMEM158 tumors relative to control tumors or U251MG-OE-TMEM158 tumors. Interestingly, we observed that the tumors of U251MG-OE-TMEM158 tumor-bearing mice exhibited an invasive border, while those in U251MG-shTMEM158 mice presented a smooth border (Fig. 6E). Moreover, we stained the intracranial tumors with TMEM158, p-STAT3, and Ki-67 antibodies. The results of IHC analysis revealed decreased TMEM158, p-STAT3, and Ki-67 levels in U251MG-shTMEM158 mice, consistent with the results of the in vitro experiments (Fig. 6E). These results demonstrated that downregulating TMEM158 inhibits the invasion and proliferation of GBM cells and prolongs the survival of GBM mice.

## Discussion

GBM is the most aggressive and lethal brain tumor and has a worse outcome in humans. The major obstacle for GBM treatment is diffuse tumor cell invasion and ensuing metastasis, which cause glioma cells migrate away from the tumor core area and escape the complete surgical resection, and partially avoid chemo- and radiotherapy, and to further cause tumor recurrence [31, 36, 37]. Therefore, new targets that regulate the invasion process are urgently needed and are actively being pursued [38, 39].

TMEM158 is a commonly known transmembrane protein that is upregulated in response to activation of the Ras pathway [17, 19]. The RTK/RAS/PI3K signaling pathway is altered in 88% of GBMs [40]. Previous studies have reported the expression and functions of TMEM158 in different tumors, including pancreatic cancer [20], ovarian cancer [19], and colorectal cancer [21]. These results suggest that TMEM158 is significantly associated with worse prognosis and that overexpression of TMEM158 increases the invasion of tumor cells. In the present study, we first found that TMEM158 expression was significantly altered in 24 cancers, especially GBM. Importantly, TMEM158 displayed the highest expression in GBM among the 31 solid cancers examined (Fig. 1A) and in glioma cell lines (data not shown, https://sites.broadinstitute.org/ccle). This indicates that TMEM158 likely plays an important role in GBM compared to other cancers.

Given the assumption that TMEM158 has a unique role in GBM, analysis of glioma sample clinical information and mRNA expression data obtained from TCGA and CGGA indicated that TMEM158 expression was related to WHO glioma grades and was more highly expressed in GBM than in lower grade gliomas (Fig. 1B–D). In 2008, the molecular era for diffuse glioma classification kicked off when isocitrate dehydrogenase 1 (*IDH1*) was reported in a small set of GBMs [41]. GBM patients who harbor a mutant form of *IDH1* were younger and exhibited a much better survival time than patients with IDH1-WT GBMs. These results are consistent with another article published several month later reporting that *IDH1* mutations occurred in more than 70% of lower-grade gliomas and secondary GBMs. In contrast, *IDH1* mutations rarely occurred in primary GBMs [42]. Importantly, wild-type *IDH1* was correlated with much worse clinical outcomes in patients with gliomas [30, 43, 44]. Our investigation indicated that TMEM158 expression was positively related to WHO glioma grade and was more highly expressed in IDH1-WT gliomas than in IDH1-Mut gliomas for each WHO classification grade. TMEM158 presented the highest expression in IDH1-WT GBMs (Figs. 1B–G, 2). Importantly, TMEM158 also offers prognostic value. Patients with lower TMEM158 expression had a much longer OS than those with higher TMEM158 expression (Fig. 1H-L). This surprising finding provided us a reliable basis for further study.

TMEM158 is associated with pro-tumor processes. Diffuse gliomas are a family of neoplastic diseases characterized by higher proliferation, motility, and angiogenesis with reduced apoptosis [45, 46]. Several studies have confirmed that overexpression of TMEM158 enhanced the growth, adhesion, and motility of tumor cells through the TGF-β and PI3K/AKT signaling pathway [19, 20]. However, the functions of TMEM158 in glioma are not clear thus far. In this study, we demonstrated that upregulating TMEM158 promotes the proliferation, migration, and invasion of glioma cells (Fig. 3). In addition, GO analysis and KEGG enrichment analysis indicated that TMEM158-high-expression genes were closely related to extracellular matrix organization, extracellular structure organization, skeletal system morphogenesis, focal adhesion, ECM-receptor interaction, and the cell cycle (Figure S1A–D). Multiple investigations identified that these processes were closely correlated with cell proliferation, invasion, and migration [47–52]. These findings are consistent with the phenotypes obtained from the cell proliferation assay, cell migration and invasion assay (Fig. 3).

The exact pathways that TMEM158 may regulate in glioma remain unclear. Therefore, we further performed GSEA and showed that TMEM158-associated genes were positively related to EMT and TNFα signaling via NF-κB and IL6-JAK-STAT3 signaling (Fig. 4A–D). EMT is a complex cellular process that allows epithelial cells to acquire a mesenchymal phenotype and is frequently observed in invasion human cancers [34, 53, 54]. The mesenchymal transition of glioma cells is correlated with a treatment-resistant and more aggressive phenotype, leading to worse outcomes and rapid progression in patients with GBMs [55–57]. Our data indicated that overexpression of TMEM158 decreases the expression of E-Ca, increases the expression of N-Ca, Snail, and vimentin and promotes the EMT process (Fig. 4E–F). Furthermore, our analysis confirmed that TMEM158 knockdown inhibits the expression of p-STAT3 (Figs. 4G-H, 6E). However, loss- or gain-of-function of TMEM158 did not affect the expression of p65 (data not shown). Many studies have reported that STAT3 transcriptionally regulates numerous downstream target genes that are crucial for tumor cell growth, migration, invasion, and immune evasion [58, 59]. Importantly, our results also demonstrated that overexpression of STAT3 rescues the TMEM158 knockdown-mediated proliferation, migration, invasion, and EMT process, indicating that TMEM158 promotes the proliferation, migration, and invasion of glioma cells by activating STAT3 signaling (Fig. 5).

Despite these findings, the current study is only a preliminary examination of TMEM158 in GBM, which has certain limitations. First, the underlying mechanism of TMEM158-mediated malignant carcinogenesis involved in STAT3 signaling remains unclear. Second, the upstream factors (such as transcription factors, cytokines, microRNAs, lncRNAs, or circRNAs) that may participant in the activation of TMEM158 in GBM are still unknown. Finally, there is currently a lack of drugs targeting TMEM158. Therefore, it is necessary to conduct more research in the future to clarify the direct upstream and downstream mechanism of TMEM158 in GBM and to develop fat-soluble, small molecule-targeted drugs targeting TMEM158 for further in vivo and in vitro studies to determine whether they have clinical translational value.

In conclusion, our work describes the molecular and clinical characterization of TMEM158 in glioma. TMEM158 is positively associated with glioma grade, IDH1 mutation status, and poor prognosis. For the first time, we associated TMEM158 with glioma cell growth, migration, and invasion via EMT and STAT3 signaling, suggesting that it may represent a useful therapeutic target for GBMs.

### Supplementary information

Supplementary Figure legends

Supplementary Figures
