# Correction: TMEM158 promotes the proliferation and migration of glioma cells via STAT3 signaling in glioblastomas

**DOI:** 10.1038/s41417-024-00733-3

**Published:** 2024-01-26

**Authors:** Jiabo Li, Xuya Wang, Lulu Chen, Jinhao Zhang, Yiming Zhang, Xiao Ren, Jinzhang Sun, Xiaoguang Fan, Jikang Fan, Tao Li, Luqing Tong, Li Yi, Lei Chen, Jie Liu, Guanjie Shang, Xiude Ren, Hao Zhang, Shengping Yu, Haolang Ming, Qiang Huang, Jun Dong, Chen Zhang, Xuejun Yang

**Affiliations:** 1 Department of Neurosurgery, Tianjin Medical University General Hospital, Tianjin, China; 2 Laboratory of Neuro-oncology, Tianjin Neurological Institute, Tianjin, China; 3 Department of Neurosurgery, The First Affiliated Hospital, Zhejiang University School of Medicine, Hangzhou, Zhejiang China; 4 Department of Neurosurgery, Union Hospital, Tongji Medical College, Huazhong University of Science and Technology, Wuhan, Hubei China; 5 Department of Neurosurgery, The Second Affiliated Hospital of Soochow University, Suzhou, Jiangsu China

**Keywords:** CNS cancer, Oncogenes, CNS cancer, Tumour biomarkers

Correction to: *Cancer Gene Therapy* 10.1038/s41417-021-00414-5, published online 06 January 2022

Following the publication of this article, reuse of an image was noted in Figures 3J and 3N depicting the migration and invasion of the sh-TMEM158-1 treated group in the U251MG cell line. Upon reviewing these figures alongside the original data presented in the article, the authors apologise for the error which occurred due to the similarity in folder names and picture titles within the original data, specifically within the same treatment group of the same cell line. Regrettably, the authors processed the migration image of the U251MG cell line sh-TMEM158-1 treated group as an invasion figure. The authors apologise for any inconvenience caused.

The corrected Figures 3J and 3N are provided below:
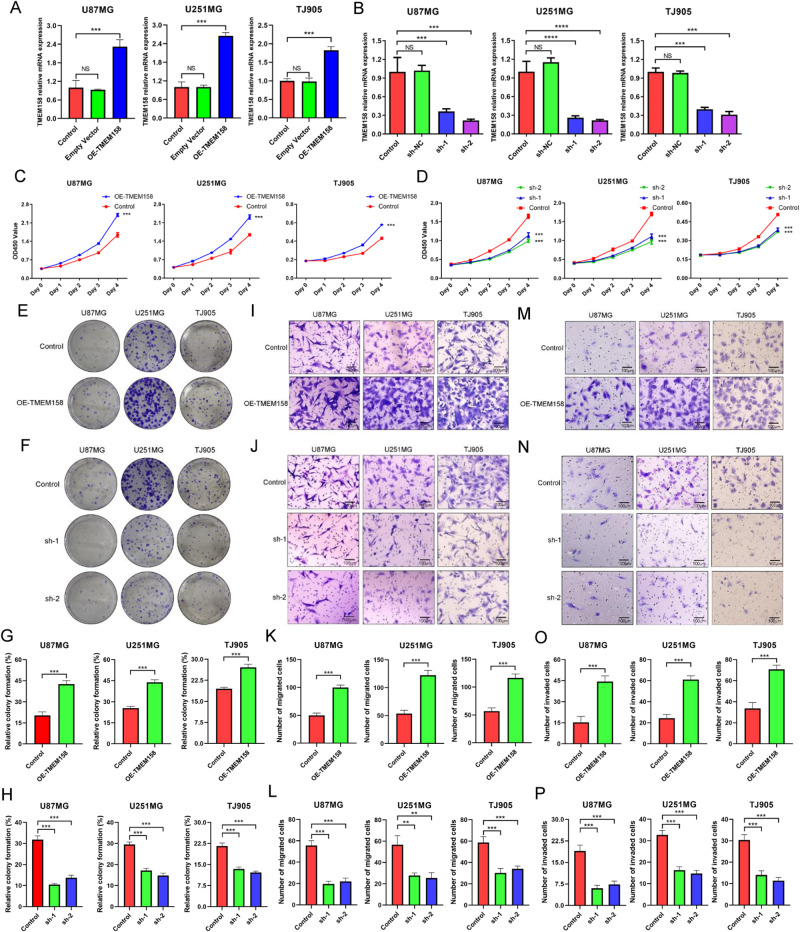


The original article has been corrected.

