# Supplementary material for: TMEM158 promotes the proliferation and migration of glioma cells via STAT3 signaling in glioblastomas
Source: Cancer Gene Ther. 2022 Jan 6;29(8-9):1117–29. doi: 10.1038/s41417-021-00414-5 (PMC9395270; doi:10.1038/s41417-021-00414-5)
Supplement: Supplementary file 1 — Supplementary Figure legends [file 41417_2021_414_MOESM1_ESM.docx]

**Supplementary Figure legends**

**Supplementary Figure 1. GO analysis and KEGG analysis of TMEM158-associated genes in glioma**

**A-B.** GO (biological process) enrichment analysis of TMEM158-Low-Expression genes and TMEM158-High-Expression genes in the TCGA database. **C-D.** KEGG pathway enrichment analysis of TMEM158-Low-Expression genes and TMEM158-High-Expression genes in the TCGA database.
