# Supplementary figures and images for: TMEM158 promotes the proliferation and migration of glioma cells via STAT3 signaling in glioblastomas
Source: Cancer Gene Ther. 2022 Jan 6;29(8-9):1117–29. doi: 10.1038/s41417-021-00414-5 (PMC9395270; doi:10.1038/s41417-021-00414-5)

**Supplementary Figure 1**

B


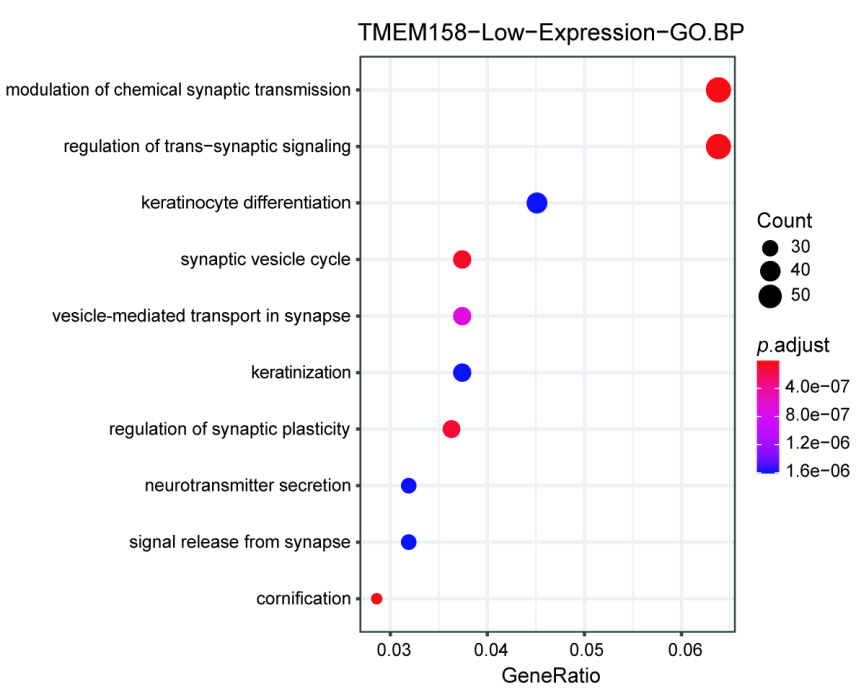

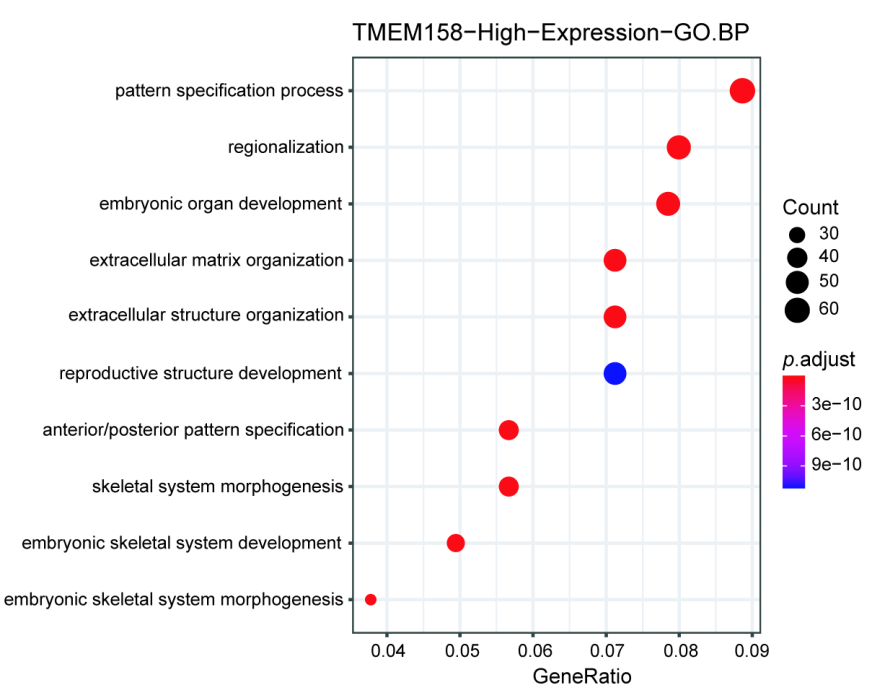


D

C

A


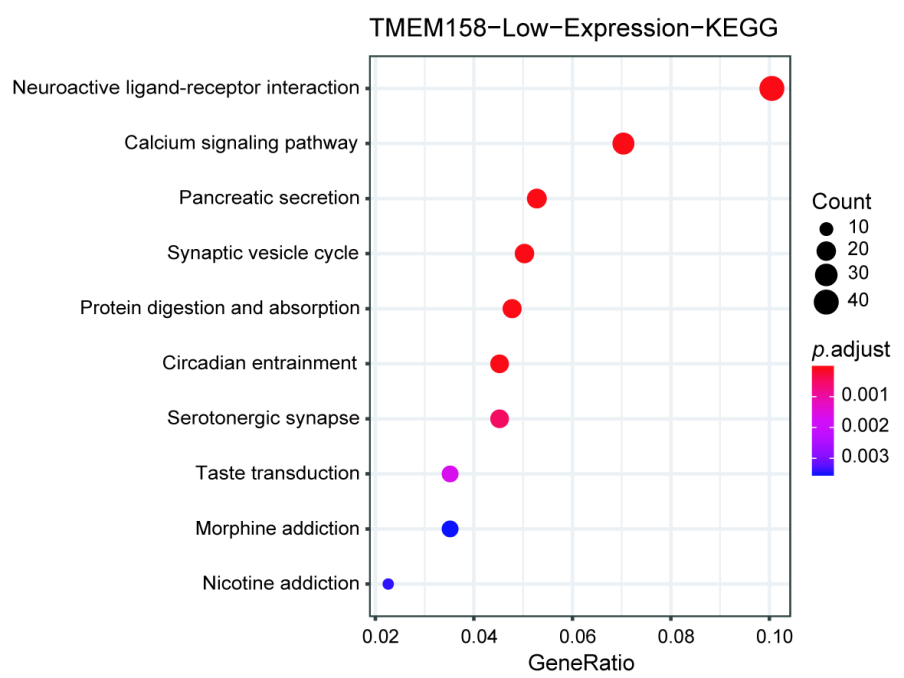

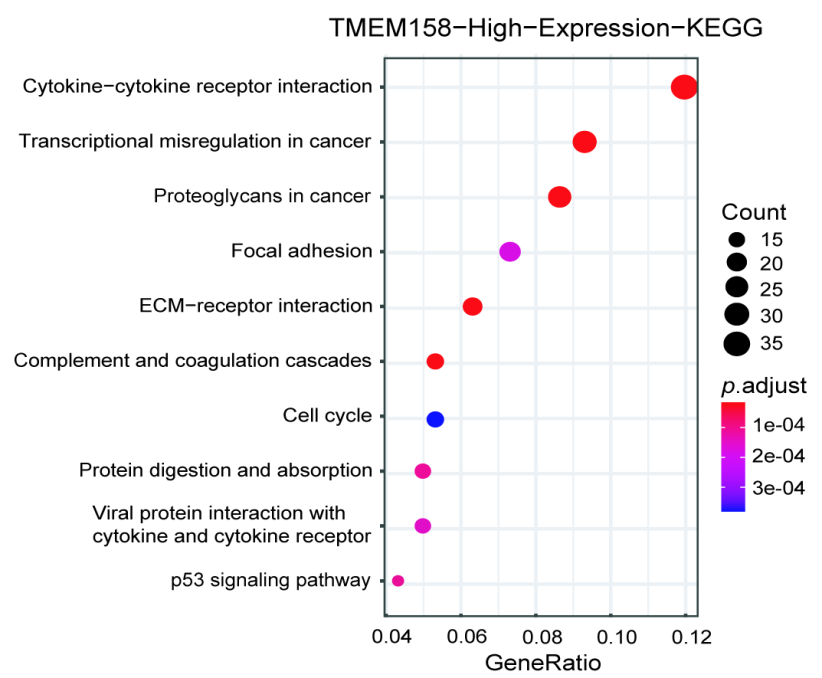

Supplement: Supplementary file 2 — Supplementary Figures [file 41417_2021_414_MOESM2_ESM.docx]
